# Supplementary material for: Anti-necroptotic effects of human Wharton’s jelly-derived mesenchymal stem cells in skeletal muscle cell death model via secretion of GRO-α
Source: PLoS One. 2024 Dec 2;19(12):e0313693. doi: 10.1371/journal.pone.0313693 (PMC11611217; doi:10.1371/journal.pone.0313693)

Raw image of western blots in Fig 2A-1

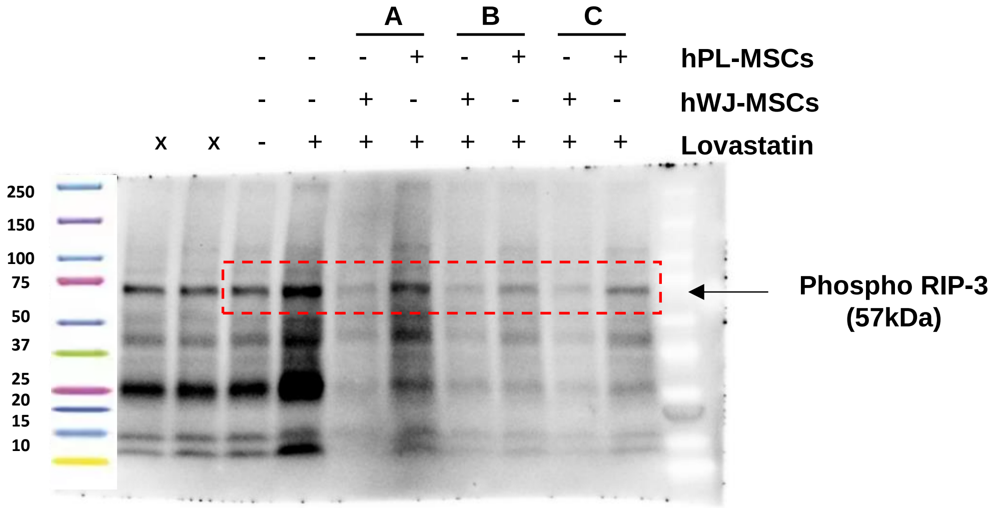

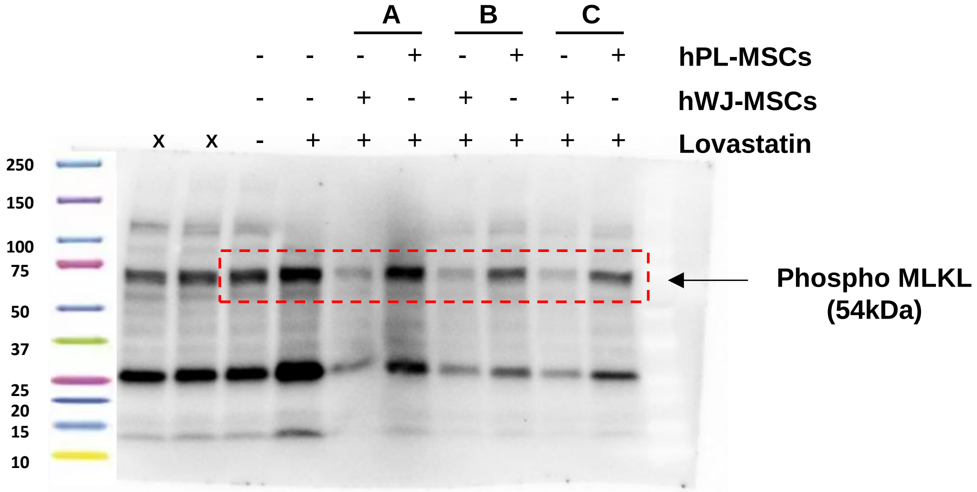

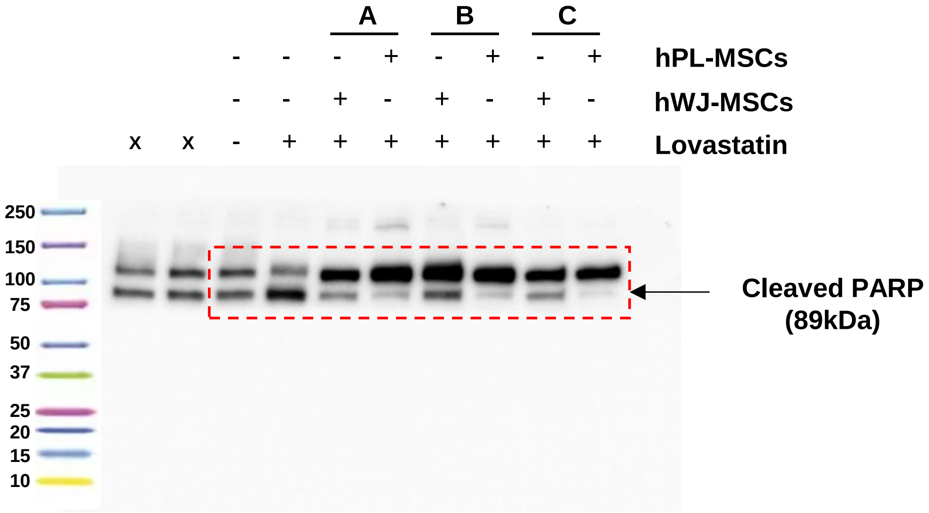

Raw image of western blots in Fig 2A-2

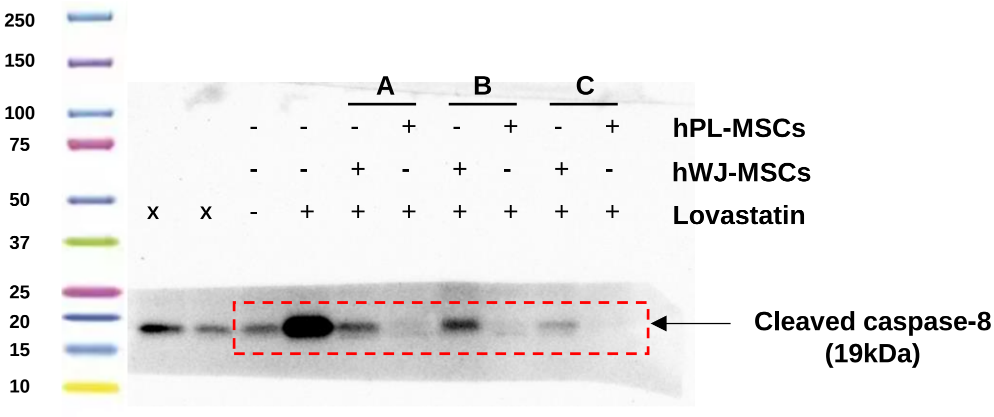

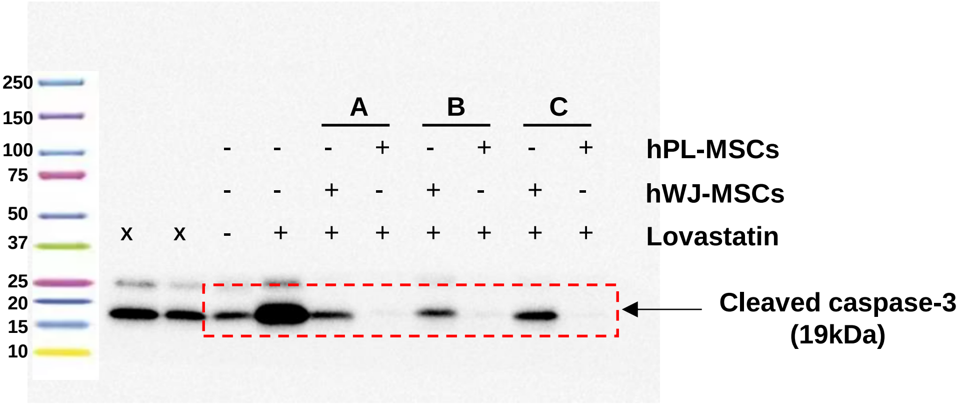

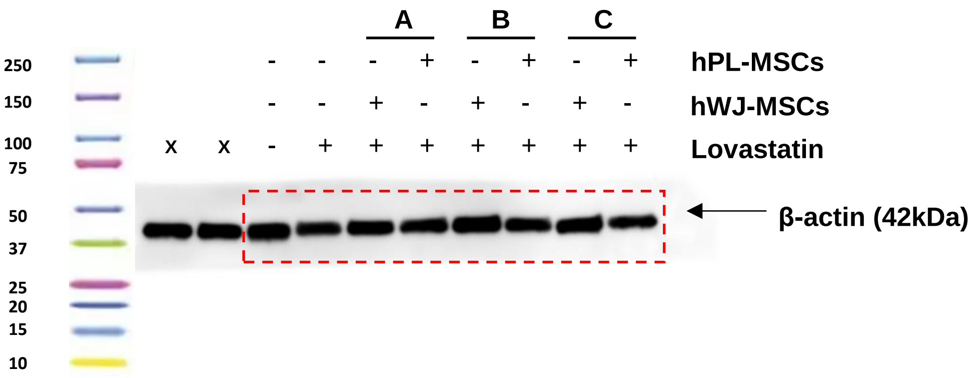

Raw image of western blots in Fig 5B

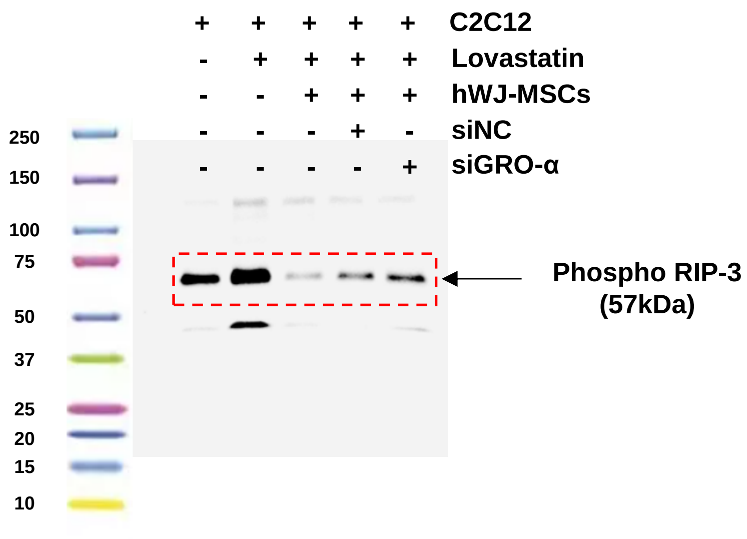

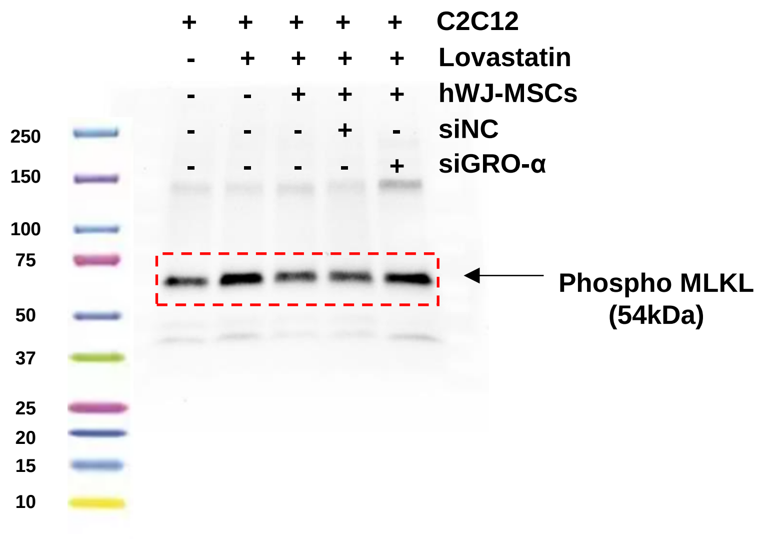

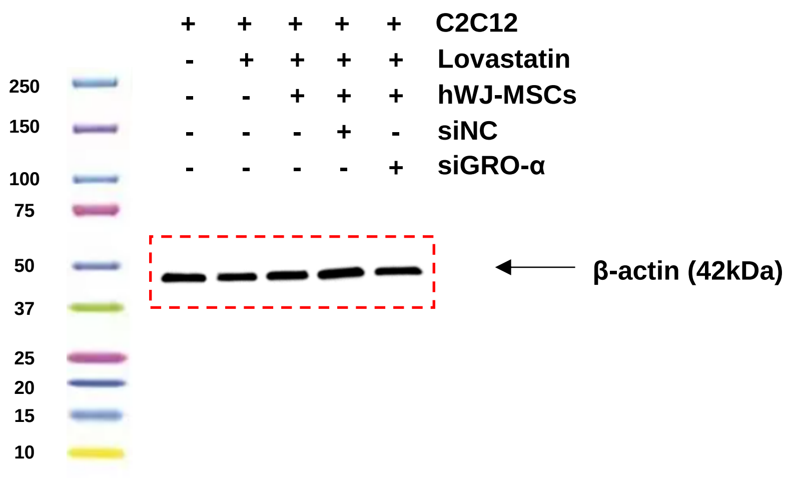

Raw image of western blots in Fig 6A

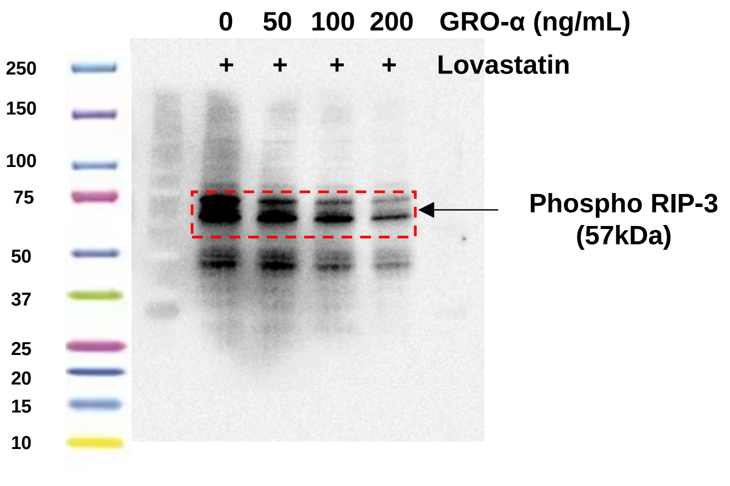

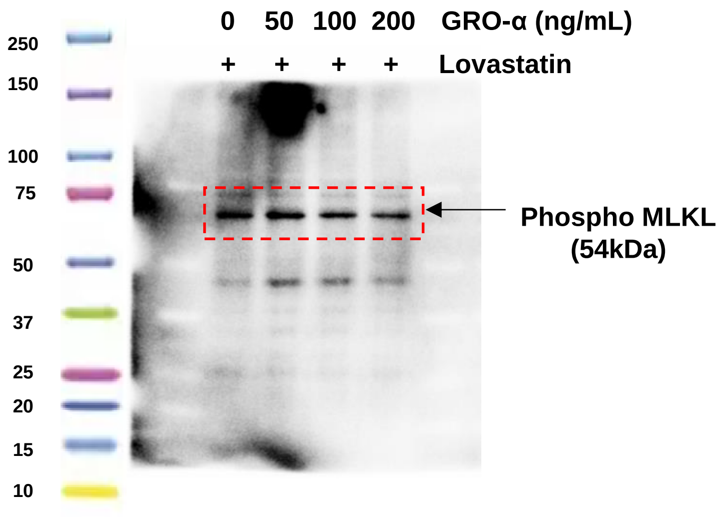

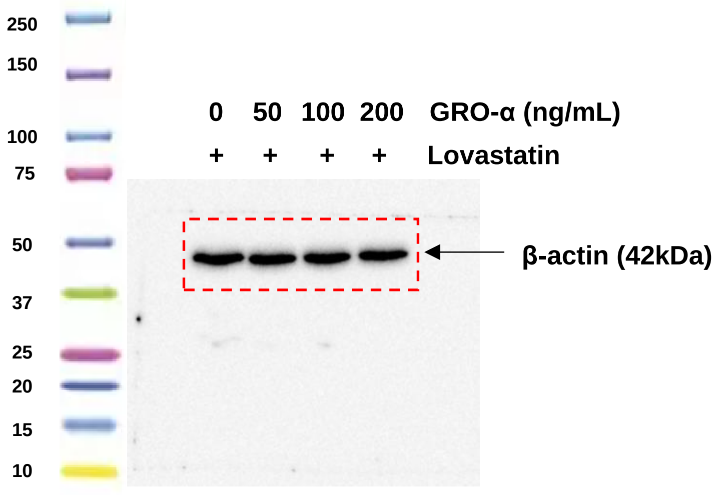

Raw image of western blots in Fig 7A

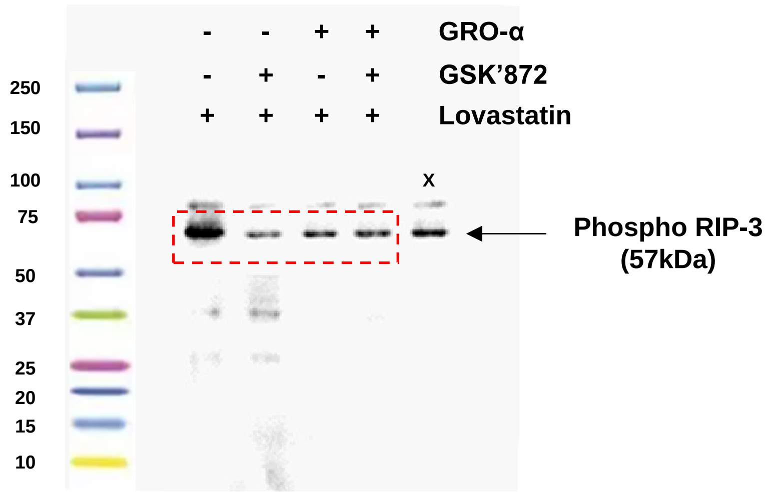

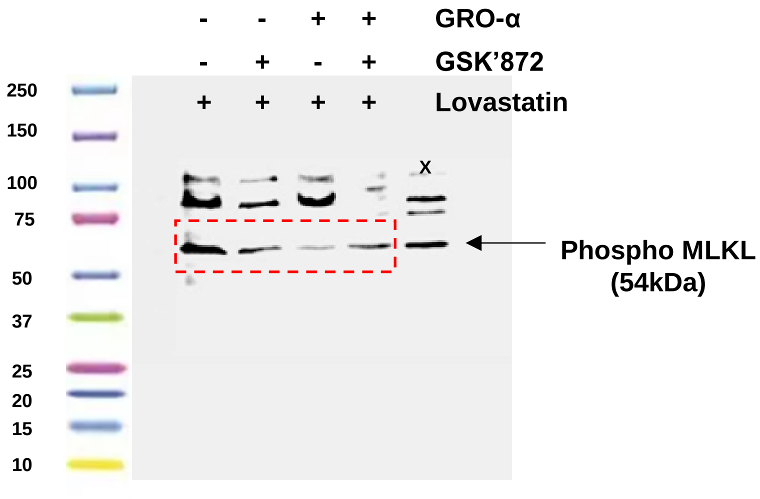

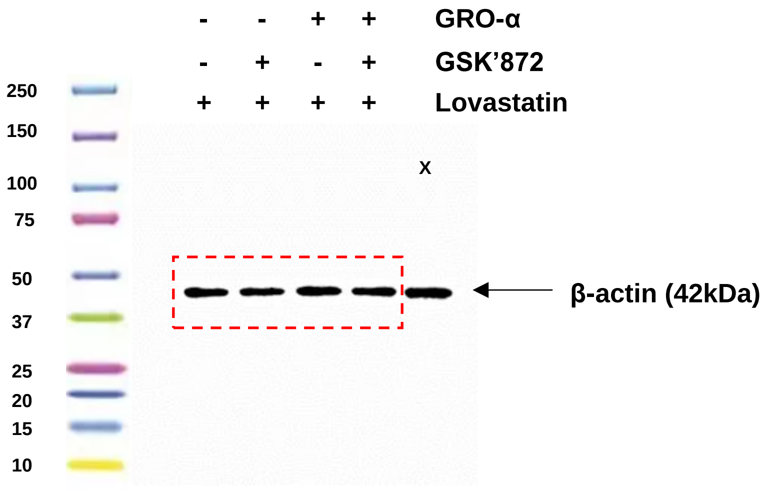

Raw image of western blots in Fig 8D

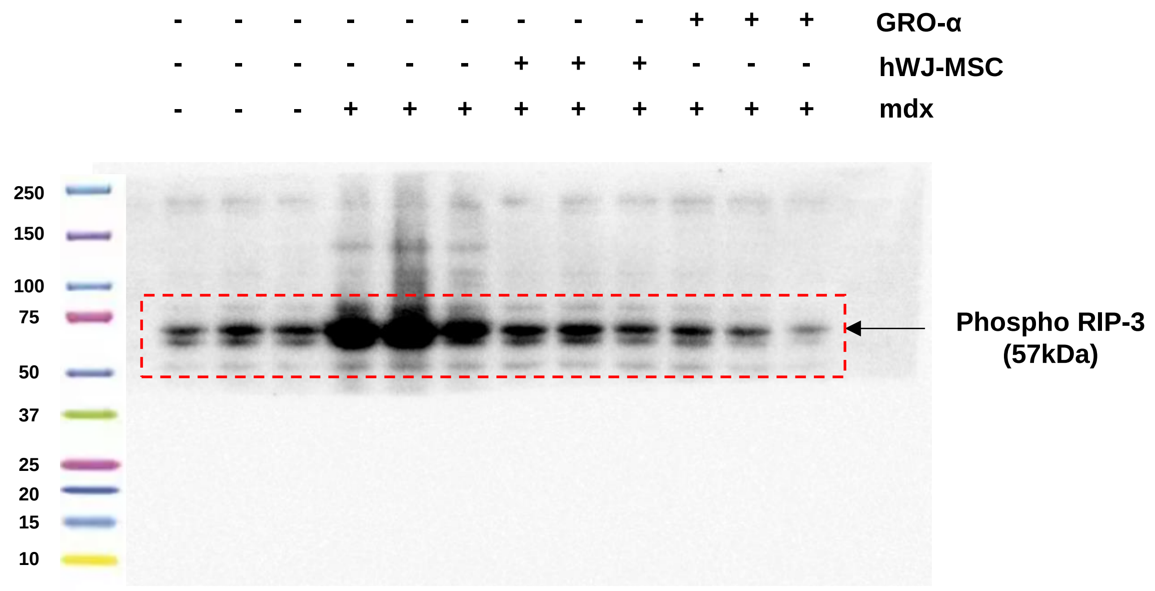

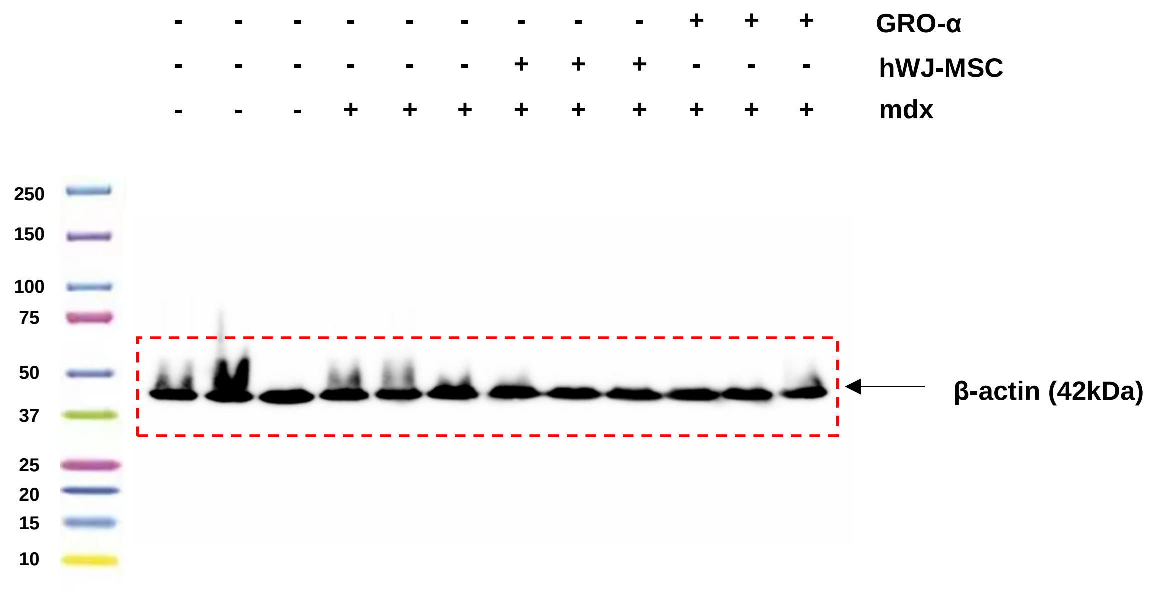

Raw image of western blots in Fig 8E

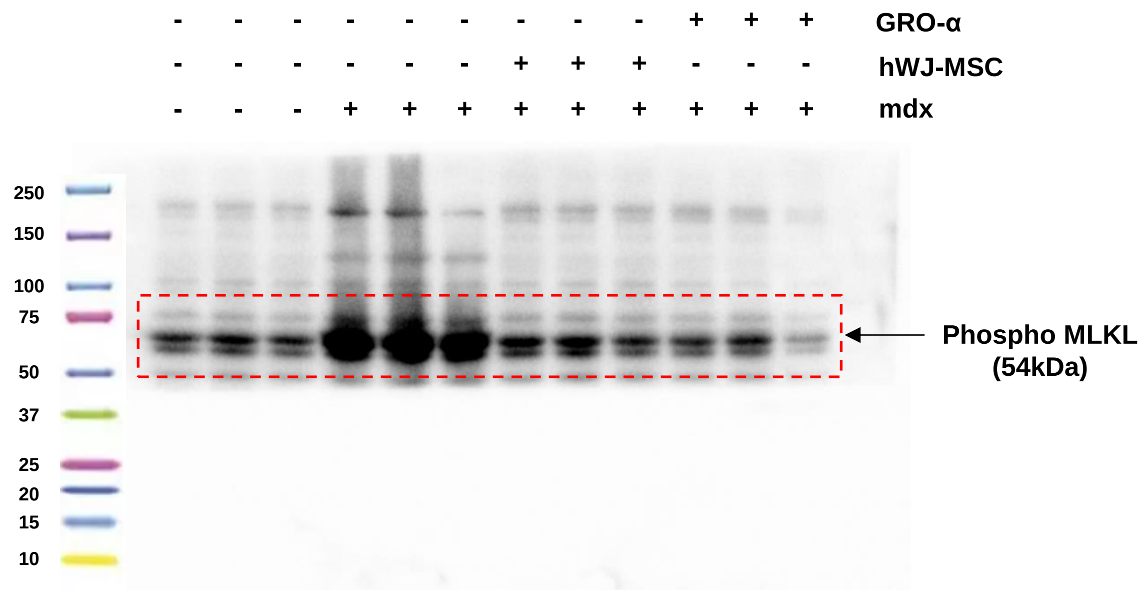

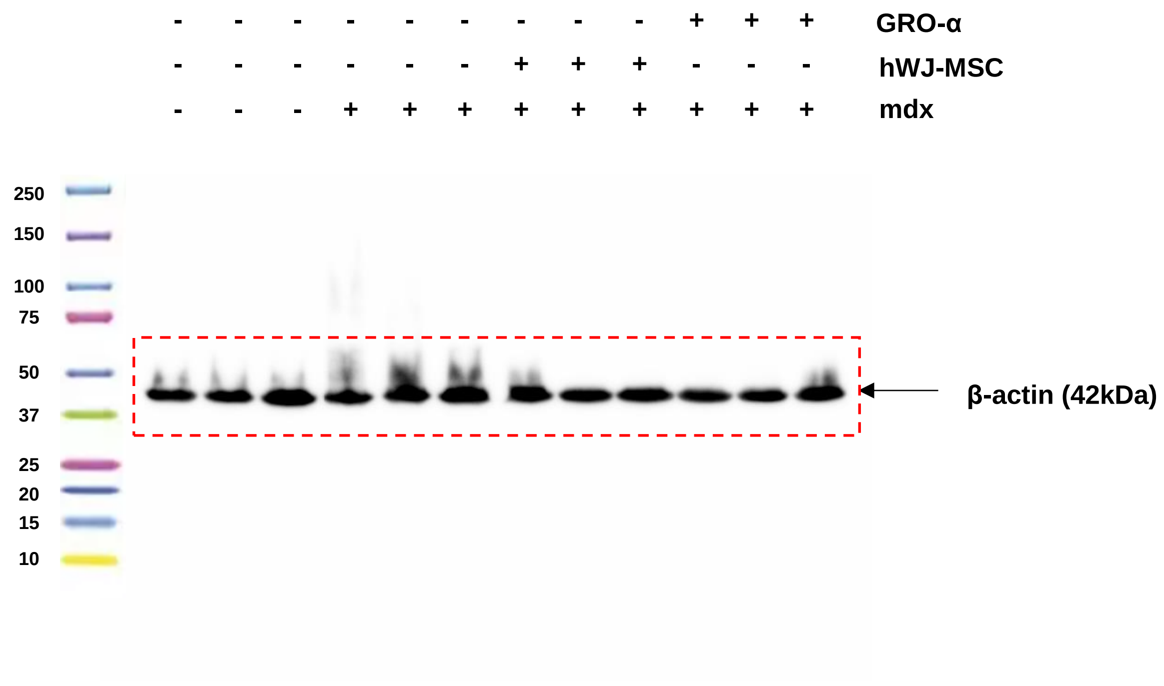

Raw image of western blots in Fig S3A

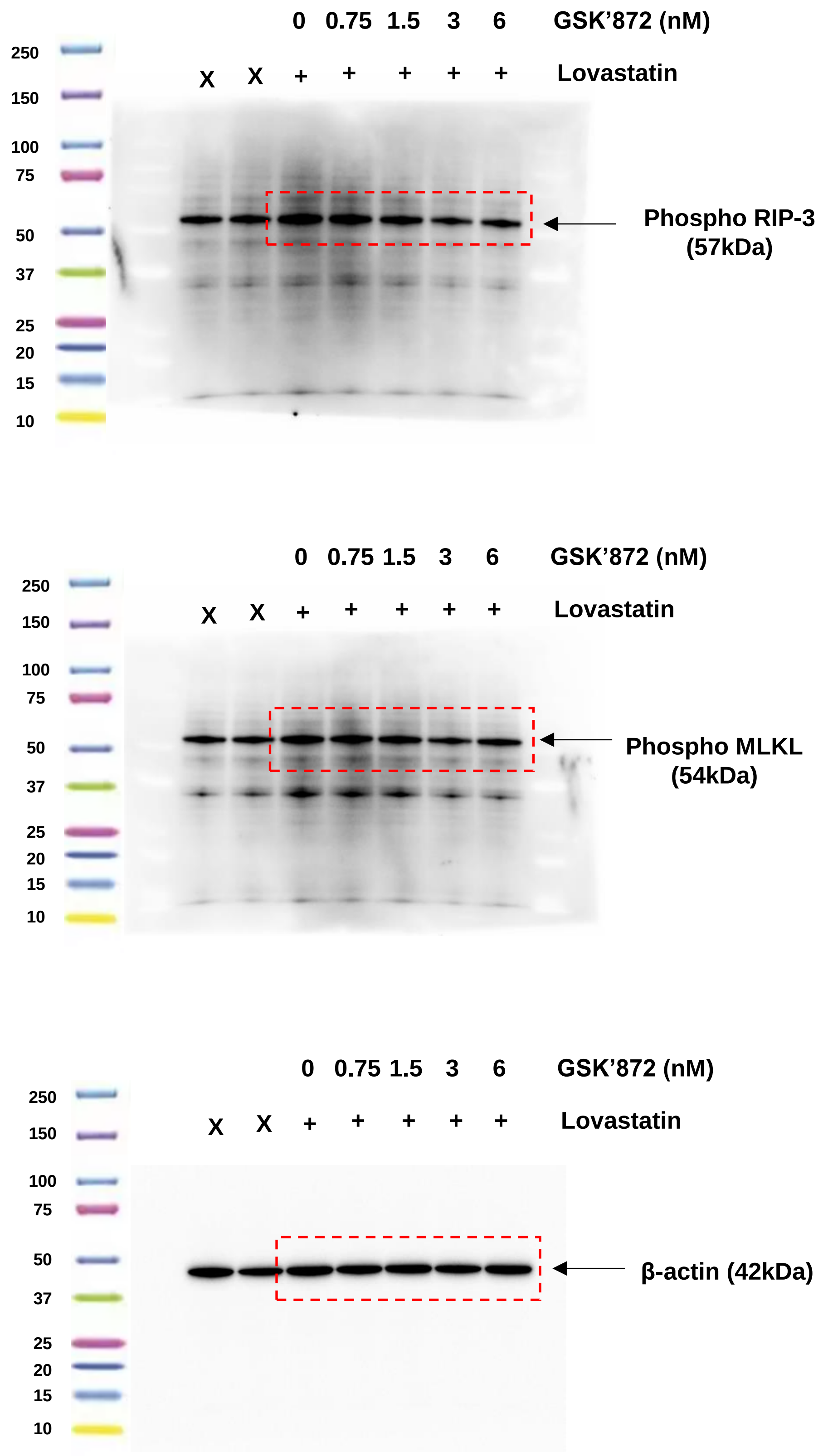

Supplement: S1 Fig — (PDF) [file pone.0313693.s001.pdf]
