# Supplementary figures and images for: Anti-necroptotic effects of human Wharton’s jelly-derived mesenchymal stem cells in skeletal muscle cell death model via secretion of GRO-α
Source: PLoS One. 2024 Dec 2;19(12):e0313693. doi: 10.1371/journal.pone.0313693 (PMC11611217; doi:10.1371/journal.pone.0313693)

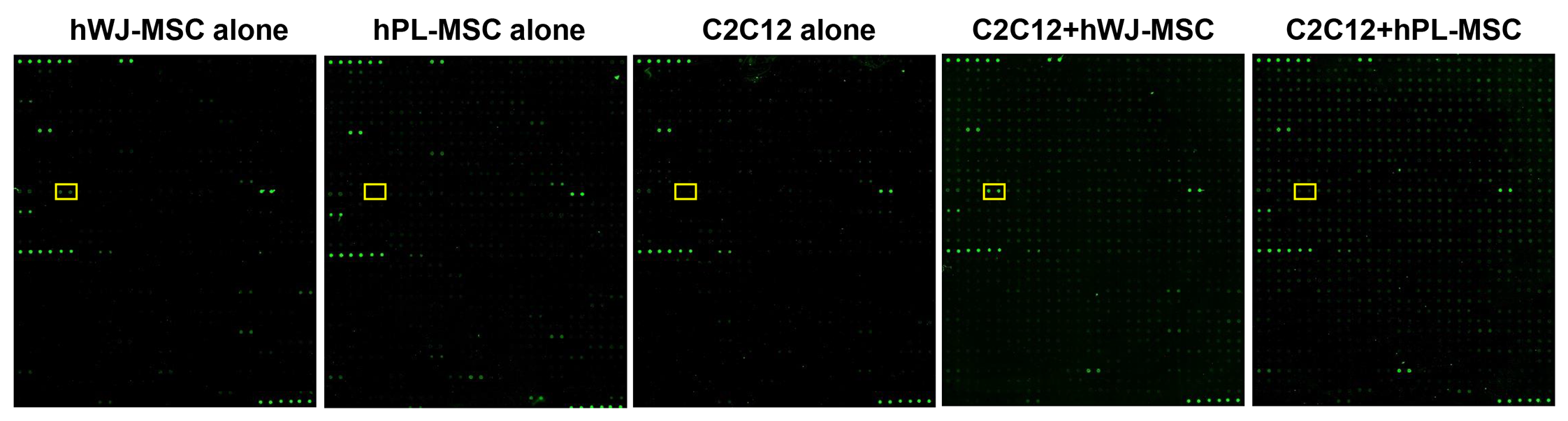

Supplement: S2 Fig — The yellow box represents growth regulated oncogene-alpha (GRO-α) expression in the culture medium under each condition. (TIF) [file pone.0313693.s002.tif]

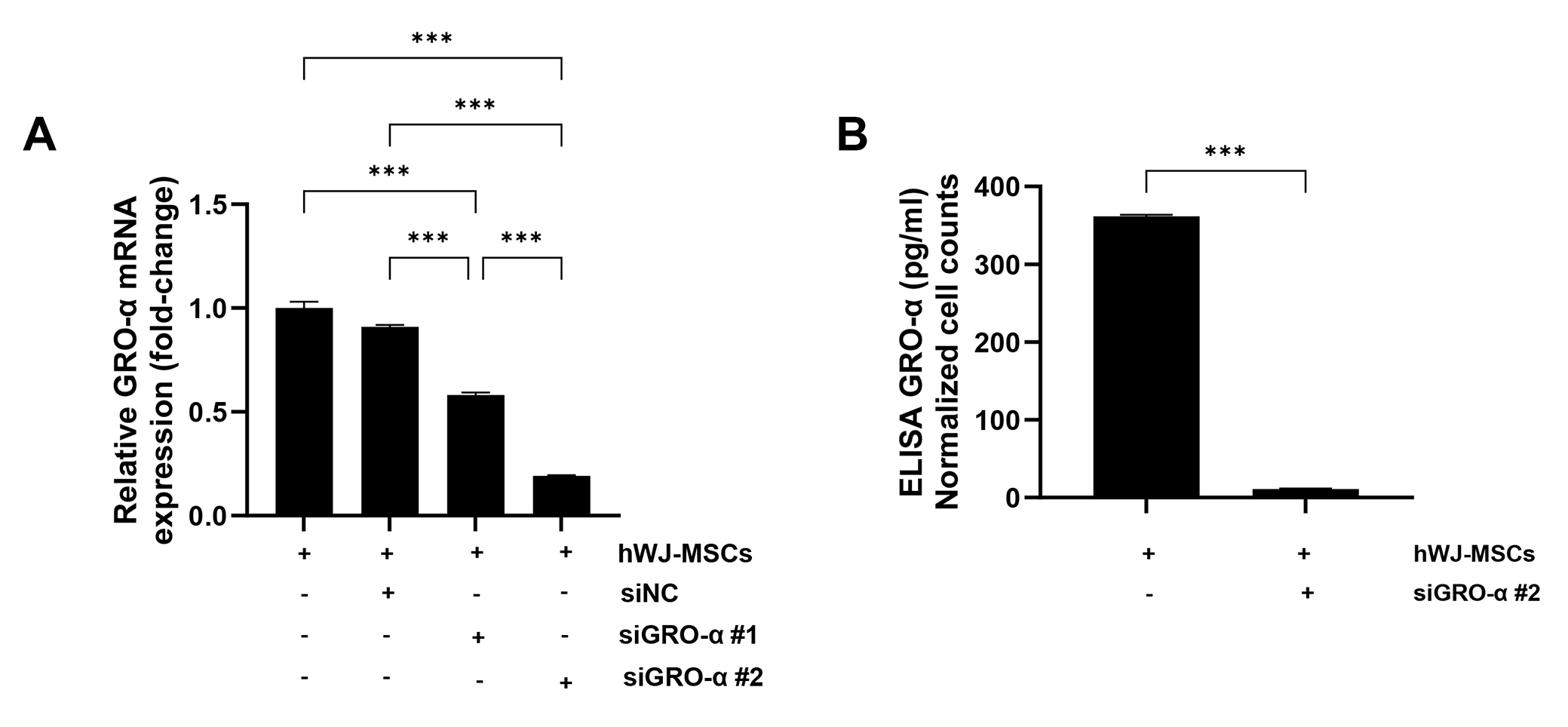

Supplement: S3 Fig — To knockdown GRO-α in hWJ-MSCs, hWJ-MSCs were pretreated with an siRNA control (siNC) or siRNA #1 and siRNA #2 against GRO-α for 24 and 48h (A, B) The GRO-α mRNA expression levels in hWJ-MSCs were measured using qRT-PCR (***p < 0.001; n = 3). (C) The GRO-α levels in each media were measured using ELISA (***p <0.001; n = 3). (TIF) [file pone.0313693.s003.tif]

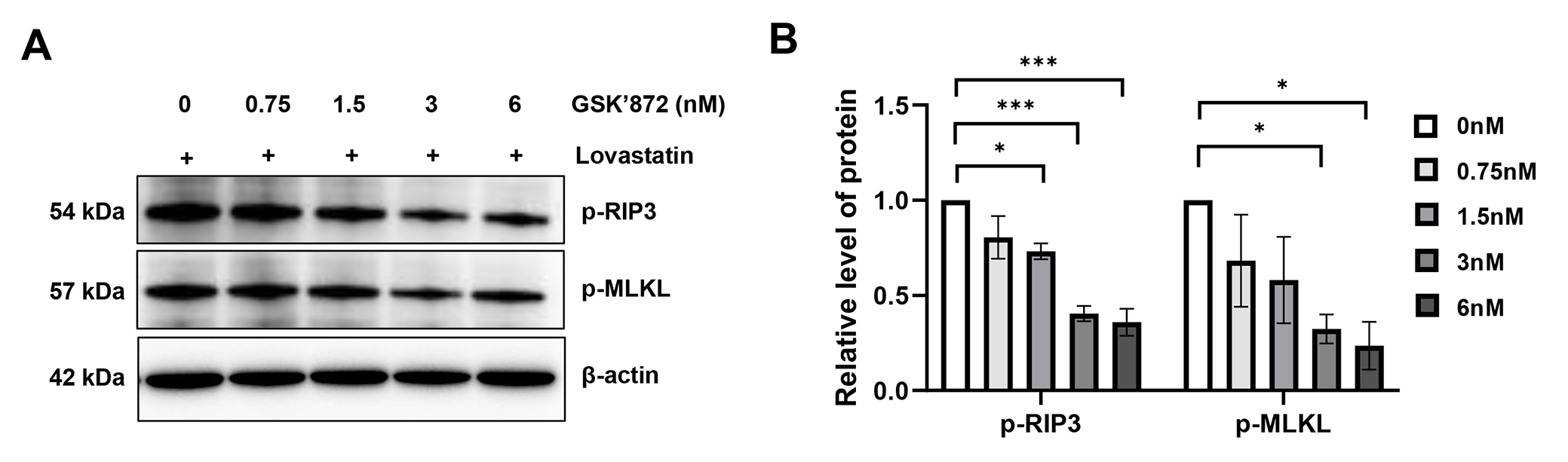

Supplement: S4 Fig — To confirm the GSK’872 concentration for anti-necrosis, the damaged C2C12 cells were treated with various GSK’872 concentrations. (A) The harvested cells were analyzed using western blotting with antibodies against p-RIP3, p-MLKL, and β-actin. (B) All bands were analyzed by densitometry (***p < 0.001, *p < 0.05, n = 3). These results were significantly different from those of the corresponding control group. (TIF) [file pone.0313693.s004.tif]
