# Supplementary material for: Anti-necroptotic effects of human Wharton’s jelly-derived mesenchymal stem cells in skeletal muscle cell death model via secretion of GRO-α
Source: PLoS One. 2024 Dec 2;19(12):e0313693. doi: 10.1371/journal.pone.0313693 (PMC11611217; doi:10.1371/journal.pone.0313693)
Supplement: S1 Table — The hMSC surface markers were identified using flow cytometry. The results for positive (CD44, CD73, CD90, and CD105) and negative (CD11b, CD14, CD19, CD34, CD45, and HLA-DR) markers are shown. (DOCX) [file pone.0313693.s005.docx]

**Supplementary Table 1. Surface marker analysis (%)**

|  | **Positive marker** | | | | **Negative Marker** | | | | | |
| --- | --- | --- | --- | --- | --- | --- | --- | --- | --- | --- |
|  | **CD44** | **CD73** | **CD90** | **CD105** | **CD11b** | **CD14** | **CD19** | **CD34** | **CD45** | **HLA-DR** |
| **WJ A** | **97.6** | **99.6** | **99.8** | **99.4** | **0.0** | **0.2** | **0.0** | **0.1** | **0.0** | **0.0** |
| **WJ B** | **99.1** | **99.8** | **100** | **99.8** | **0.0** | **0.0** | **0.0** | **0.0** | **0.0** | **0.0** |
| **WJ C** | **99.9** | **99.9** | **100** | **99.9** | **0.1** | **0.1** | **0.0** | **0.0** | **0.0** | **0.0** |
| **PL A** | **99.8** | **100** | **92.6** | **100** | **0.0** | **0.1** | **0.0** | **0.0** | **0.0** | **0.0** |
| **PL B** | **100** | **99.9** | **94.9** | **99.9** | **0.0** | **0.1** | **0.0** | **0.0** | **0.0** | **0.0** |
| **PL C** | **100** | **100** | **98.6** | **100** | **0.0** | **0.1** | **0.0** | **0.0** | **0.0** | **0.0** |
