# Supplementary material for: Anti-necroptotic effects of human Wharton’s jelly-derived mesenchymal stem cells in skeletal muscle cell death model via secretion of GRO-α
Source: PLoS One. 2024 Dec 2;19(12):e0313693. doi: 10.1371/journal.pone.0313693 (PMC11611217; doi:10.1371/journal.pone.0313693)
Supplement: S2 Table — (DOCX) [file pone.0313693.s006.docx]

**Supplementary Table 2. Doubling Time (hrs)**

|  | P1 | P2 | P3 | P4 |
| --- | --- | --- | --- | --- |
| **WJ A** | 54.46 | 91.09 | 42.75 | 43.92 |
| **WJ B** | 88.91 | 58.74 | 31.36 | 12.17 |
| **WJ C** | 30.26 | 29.83 | 28.56 | 41.34 |
| **PL A** | 25.29 | 36.59 | 36.17 | 31.46 |
| **PL B** | 73.56 | 54.37 | 41.74 | 57.20 |
| **PL C** | 37.67 | 42.69 | 27.61 | 33.59 |
