# Supplementary material for: Anti-necroptotic effects of human Wharton’s jelly-derived mesenchymal stem cells in skeletal muscle cell death model via secretion of GRO-α
Source: PLoS One. 2024 Dec 2;19(12):e0313693. doi: 10.1371/journal.pone.0313693 (PMC11611217; doi:10.1371/journal.pone.0313693)
Supplement: S3 Table — (DOCX) [file pone.0313693.s007.docx]

**Supplementary Table 3. Population doubling level (PDL)**

|  | P1 | P2 | P3 | P4 |
| --- | --- | --- | --- | --- |
| **WJ A** | 2.55 | 1.30 | 3.46 | 2.55 |
| **WJ B** | 1.33 | 2.38 | 3.76 | 4.11 |
| **WJ C** | 3.27 | 3.12 | 3.19 | 2.27 |
| **PL A** | 3.52 | 2.54 | 2.60 | 2.92 |
| **PL B** | 2.22 | 2.70 | 3.33 | 2.05 |
| **PL C** | 3.66 | 3.44 | 3.22 | 2.17 |
