# Supplementary material for: Anti-necroptotic effects of human Wharton’s jelly-derived mesenchymal stem cells in skeletal muscle cell death model via secretion of GRO-α
Source: PLoS One. 2024 Dec 2;19(12):e0313693. doi: 10.1371/journal.pone.0313693 (PMC11611217; doi:10.1371/journal.pone.0313693)
Supplement: Attachment — Submitted filename: Response to Reviewers.docx [file pone.0313693.s008.docx]

September 25th, 2024

**Atsushi Asakura, Ph.D**

Academic Editor

*PLOS ONE*

Dear Dr. Asakura,

Please find enclosed the revised version of Manuscript ID PONE-D-24-11604, “**Anti-necroptotic effects of human Wharton’s jelly-derived mesenchymal stem cells in skeletal muscle cell death model via secretion of GRO-α**” and our point-by-point responses to the reviewers’ comments. We have carefully considered the points that were raised by the reviewers and have made all the requested changes.

Please note that we have used red font instead of black to indicate the revised portions of our manuscript. We greatly appreciate the insightful comments made by the reviewer and believe that reviewer’s comments have contributed towards improving the overall quality of our manuscript. We hope that you will approve of our revisions and the current version of our manuscript to be suitable for publication in *PLOS ONE.*

Thank you for your consideration. We look forward to hearing from you.

Sincerely,

**Jong Wook Chang, PhD**

Department of Health Sciences and Technology, SAIHST, Sungkyunkwan University, Seoul 06355, Republic of Korea

Tel: +82-2-3410-6048; Fax: +82-2-6205-8054; Email: jongwook.chang@samsung.com

**Hong Bae Jeon, PhD**

Cell and Gene Therapy Institute, ENCell Co. Ltd., Seoul 06072, Republic of Korea

Tel: +82-2-6205-8055; Fax: +82-2-6205-8054; Email: jhb@encellinc.com

PONE-D-24-11604R1
Anti-necroptotic effects of human Wharton’s jelly-derived mesenchymal stem cells in skeletal muscle cell death model via secretion of GRO-α
Research Director Hong Bae Jeon

Dear Dr. Jeon,

We've checked your submission and before we can proceed, we need you to address the following issues:

1. In your Data Availability statement you say that the antibody array data generated in the present study can be found in the GEO under accession number GSE277126 (https://www.ncbi.nlm.nih.gov/geo/query/acc.cgi?acc=GSE277126). Accession "GSE277126" is currently private and is scheduled to be released on Dec 31, 2024. Can you update this so that the data can be viewed.

Reply: The antibody array data generated in the present study can be found in the GEO under the accession number GSE277126 (https://www.ncbi.nlm.nih.gov/geo/query/acc.cgi?acc=GSE277126). As your request, we have updated the data release date to September 25, 2024.

View Letter

Date:	Sep 06 2024 04:57AM

To:	"Hong Bae Jeon" jhb@encellinc.com

From:	"PLOS ONE" plosone@plos.org

Subject:	PLOS ONE Decision: Revision required [PONE-D-24-11604]

PONE-D-24-11604

Anti-necroptotic effects of human Wharton’s jelly-derived mesenchymal stem cells in skeletal muscle cell death model via secretion of GRO-α

PLOS ONE

Dear Dr. Jeon,

Thank you for submitting your manuscript to PLOS ONE. After careful consideration, we feel that it has merit but does not fully meet PLOS ONE’s publication criteria as it currently stands. Therefore, we invite you to submit a revised version of the manuscript that addresses the points raised during the review process.

Please submit your revised manuscript by Oct 21 2024 11:59PM. If you will need more time than this to complete your revisions, please reply to this message or contact the journal office at <a href="mailto:plosone@plos.org">plosone@plos.org. When you're ready to submit your revision, log on to https://www.editorialmanager.com/pone/ and select the 'Submissions Needing Revision' folder to locate your manuscript file.

Please include the following items when submitting your revised manuscript:

•	A rebuttal letter that responds to each point raised by the academic editor and reviewer(s). You should upload this letter as a separate file labeled 'Response to Reviewers'.

•	A marked-up copy of your manuscript that highlights changes made to the original version. You should upload this as a separate file labeled 'Revised Manuscript with Track Changes'.

•	An unmarked version of your revised paper without tracked changes. You should upload this as a separate file labeled 'Manuscript'.

If you would like to make changes to your financial disclosure, please include your updated statement in your cover letter. Guidelines for resubmitting your figure files are available below the reviewer comments at the end of this letter.

If applicable, we recommend that you deposit your laboratory protocols in protocols.io to enhance the reproducibility of your results. Protocols.io assigns your protocol its own identifier (DOI) so that it can be cited independently in the future. For instructions see: https://journals.plos.org/plosone/s/submission-guidelines#loc-laboratory-protocols. Additionally, PLOS ONE offers an option for publishing peer-reviewed Lab Protocol articles, which describe protocols hosted on protocols.io. Read more information on sharing protocols at https://plos.org/protocols?utm_medium=editorial-email&utm_source=authorletters&utm_campaign=protocols.

We look forward to receiving your revised manuscript.

Kind regards,

Atsushi Asakura, Ph.D

Academic Editor

PLOS ONE

Journal requirements:

1. When submitting your revision, we need you to address these additional requirements.

Please ensure that your manuscript meets PLOS ONE's style requirements, including those for file naming. The PLOS ONE style templates can be found at

https://journals.plos.org/plosone/s/file?id=wjVg/PLOSOne_formatting_sample_main_body.pdf and

https://journals.plos.org/plosone/s/file?id=ba62/PLOSOne_formatting_sample_title_authors_affiliations.pdf.

 Reply: We have modified our manuscript to meet *PLOS ONE*’s style requirements, including those for file naming.

2. PLOS ONE now requires that authors provide the original uncropped and unadjusted images underlying all blot or gel results reported in a submission’s figures or Supporting Information files. This policy and the journal’s other requirements for blot/gel reporting and figure preparation are described in detail at https://journals.plos.org/plosone/s/figures#loc-blot-and-gel-reporting-requirements and https://journals.plos.org/plosone/s/figures#loc-preparing-figures-from-image-files. When you submit your revised manuscript, please ensure that your figures adhere fully to these guidelines and provide the original underlying images for all blot or gel data reported in your submission. See the following link for instructions on providing the original image data: https://journals.plos.org/plosone/s/figures#loc-original-images-for-blots-and-gels.

In your cover letter, please note whether your blot/gel image data are in Supporting Information or posted at a public data repository, provide the repository URL if relevant, and provide specific details as to which raw blot/gel images, if any, are not available. Email us at plosone@plos.org if you have any questions.

 Reply: According to the journal’s request, we have provided the original uncropped and unadjusted images underlying all blot or gel results reported in the Supporting Information file S4 Fig. In addition, we have also highlighted this in the cover letter.

2. PLOS requires an ORCID iD for the corresponding author in Editorial Manager on papers submitted after December 6th, 2016. Please ensure that you have an ORCID iD and that it is validated in Editorial Manager. To do this, go to ‘Update my Information’ (in the upper left-hand corner of the main menu), and click on the Fetch/Validate link next to the ORCID field. This will take you to the ORCID site and allow you to create a new iD or authenticate a pre-existing iD in Editorial Manager.

 Reply: We have authenticated and added the ORCID iD of the corresponding author.

3. We note that the grant information you provided in the ‘Funding Information’ and ‘Financial Disclosure’ sections do not match.

 When you resubmit, please ensure that you provide the correct grant numbers for the awards you received for your study in the ‘Funding Information’ section.

 Reply: To match ‘Funding information’ and ‘Financial disclosure’, we have included the changed financial disclosures in the cover letter and provided the correct grant numbers for the awards we received for our study in the ‘Funding information’ section.

4. We note that you have included the phrase “data not shown” in your manuscript. Unfortunately, this does not meet our data sharing requirements. PLOS does not permit references to inaccessible data. We require that authors provide all relevant data within the paper, Supporting Information files, or in an acceptable, public repository. Please add a citation to support this phrase or upload the data that corresponds with these findings to a stable repository (such as Figshare or Dryad) and provide and URLs, DOIs, or accession numbers that may be used to access these data. Or, if the data are not a core part of the research being presented in your study, we ask that you remove the phrase that refers to these data.

 Reply: The results have been submitted as Supporting Information (S3 Fig).

5. Your ethics statement should only appear in the Methods section of your manuscript. If your ethics statement is written in any section besides the Methods, please move it to the Methods section and delete it from any other section. Please ensure that your ethics statement is included in your manuscript, as the ethics statement entered into the online submission form will not be published alongside your manuscript.

 Reply: We have moved the ethics statement to the Materials and methods section of the manuscript.

6. Please remove your figures from within your manuscript file, leaving only the individual TIFF/EPS image files, uploaded separately. These will be automatically included in the reviewers’ PDF.

 Reply: We have deleted the figures in the manuscript and uploaded the individual TIFF image files separately.

7. Please include captions for your all Supporting Information files at the end of your manuscript, and update any in-text citations to match accordingly. Please see our Supporting Information guidelines for more information: http://journals.plos.org/plosone/s/supporting-information.

Reply: Referring to the Support Information Guidelines, we have included captions of all support information files at the end of the manuscript and updated the in-text citations to match them accordingly.

[Note: HTML markup is below. Please do not edit.]

Reviewer's Responses to Questions

**Comments to the Author**

1. Is the manuscript technically sound, and do the data support the conclusions?

The manuscript must describe a technically sound piece of scientific research with data that supports the conclusions. Experiments must have been conducted rigorously, with appropriate controls, replication, and sample sizes. The conclusions must be drawn appropriately based on the data presented.

Reviewer #1: Yes

2. Has the statistical analysis been performed appropriately and rigorously?

Reviewer #1: Yes

3. Have the authors made all data underlying the findings in their manuscript fully available?

The [PLOS Data policy](http://www.plosone.org/static/policies.action#sharing) requires authors to make all data underlying the findings described in their manuscript fully available without restriction, with rare exception (please refer to the Data Availability Statement in the manuscript PDF file). The data should be provided as part of the manuscript or its supporting information, or deposited to a public repository. For example, in addition to summary statistics, the data points behind means, medians and variance measures should be available. If there are restrictions on publicly sharing data—e.g. participant privacy or use of data from a third party—those must be specified.

Reviewer #1: Yes

4. Is the manuscript presented in an intelligible fashion and written in standard English?

PLOS ONE does not copyedit accepted manuscripts, so the language in submitted articles must be clear, correct, and unambiguous. Any typographical or grammatical errors should be corrected at revision, so please note any specific errors here.

Reviewer #1: Yes

5. Review Comments to the Author

Please use the space provided to explain your answers to the questions above. You may also include additional comments for the author, including concerns about dual publication, research ethics, or publication ethics. (Please upload your review as an attachment if it exceeds 20,000 characters)

Reviewer #1: PONE-D-24-11604

Comments to the Authors

There is a sizeable literature covering the impact of site of origin of human mesenchymal stem cells (hMSCs) on their properties and potential as therapeutic agents for multiple conditions. The Authors have extended their work in this area by comparing the effects of h-MSCs isolated from placenta and Wharton’s jelly from the same individuals on in vivo and in vitro models of skeletal muscle cell death. They find that secretions from hWJ-MSCs were highly effective in inhibiting necroptosis and provide evidence suggesting that the primary mediator of this effect is GROα release by hWJ-MSCs.

Reply: We appreciate the reviewer’s constructive review of the manuscript. We believe that the reviewer has raised an important issue and agree with this comment.

General comments.
1. In the current manuscript the Authors report that when hMSCs are cultured alone, GROα is not detectable in the media, yet in their previous work (ref #27, Fig. 7e) it appears that solo-cultured hMSCs release appreciable amounts of CXCL1/GROα. This apparent discrepancy needs to be discussed; might it be due to variability between donors? Or the sensitivity of the detection methodology? It is an important point, as it impacts their hypothesis that some aspect of the microenvironment of challenged C2C12 cells is required to induce GROα secretion by hWJ-MSCs.

Reply: Thank you for your comment. We apologize for the confusion caused by what we wrote. We have added Fig. S1 for a clear understanding of the content. As shown in the figure, fluorescence in the antibody array was hardly detected in C2C12 alone or hPL-MSCs alone. Unfortunately, the part that was not detected in the hWJ-MSC only is a typo. As shown in Fig. S2B, GROα secretion was confirmed in hWJ-MSC alone. Therefore, we have modified this sentence accordingly.

Line 312-313. On the contrary, when culturing C2C12 or hPL-MSCs alone, GRO-α secretion was hardly observed (S1 Fig).

Specific comments
1. Abstract, L.40-41. The first mention of GROα is somewhat confusing. It might be better to move the sentence starting “GROα was differentially….” (L.43) to earlier in the abstract, setting up why a focus was applied to GROα.

Reply: We agree with the reviewer’s comment and have revised the sentence accordingly.

Line 25-37. Human mesenchymal stem cells (hMSCs) have therapeutic applications and potential for use in regenerative medicine. However, the use of hMSCs in research and clinical medicine is limited by a lack of information pertaining to their donor-specific functional attributes. In this study, we compared the characteristics of same-donor derived placenta (PL) and Wharton’s jelly (WJ)-derived hMSCs, we also compared their mechanism of action in a skeletal muscle disease *in vitro* model. The same-donor-derived hWJ- and hPL-MSCs exhibited typical hMSC characteristics. However, GRO-α was differentially expressed in hWJ- and hPL-MSCs. hWJ-MSCs, which secreted a high amount of GRO-α, displayed a higher ability to inhibit necroptosis in skeletal muscle cells than hPL-MSCs. This demonstrates the anti-necroptotic therapeutic effect of GRO-α in the skeletal muscle cell death model. Furthermore, GRO-α also exhibited the anti-necroptotic effect in a Duchenne muscular dystrophy (DMD) mouse model. Considering their potential to inhibit necroptosis in skeletal muscle cells, hWJ-MSCs and the derived GRO-α are novel treatment options for skeletal muscle diseases such as DMD.

2. Section 2.2, Co-culture. The sequence of these events is somewhat unclear. Were C2C12 cells reseeded after exposure to lovastatin (L. 94)? Was lovastatin still present during co-culture?

Reply: We revised the sentence to clarify the content. The modified part now reads as follows:

Line 85-90. When C2C12 reached 80–90% confluency after seeding on a six-well plate (BD Falcon, Franklin Lakes, NJ, USA), they were treated with 10 μM lovastatin for 3 h to induce cell death and washed with PBS to remove lovastatin. hWJ- and hPL-MSCs were trypsinized, suspended in serum-free MEM alpha, seeded (1×10^5^/1 mL) in 1-µm-pore-size transwell inserts (BD Biosciences, Franklin Lakes, NJ, USA), and co-cultured with C2C12 for 24 h.

3. There is some confusion about the method(s?) of euthanasia. Was it isoprene (l.114), or CO2 (L.116-119)?

Reply: We apologize for the confusion regarding the euthanasia method used. Mice were euthanized using carbon dioxide, not isoprene. So, we deleted the sentence “The mice were euthanized using isoprene, 7 days after administration”.

4. L.129. Please provide a reference for the cell counting methodology.

Reply: As you advised, we have provided a reference for the cell counting methodology.

Line 119-120. The viability of C2C12 cells was measured using the Cell Counting kit-8 (CCK-8, Dojindo, Tokyo, Japan), as previously described [30].

5. Section 2.814. Please provide catalog numbers for the antibodies used.

Reply: As you requested, we provided catalog numbers for antibodies used. The modified lines are as follows:

Line 185-191. The following primary antibodies were used: poly ADP ribose polymerase (9542L, PARP; polyclonal, Cell Signaling Technology, Danvers, MA, USA), phosphoreceptor interacting protein kinase 3 (57220S, P-RIP3; polyclonal, Cell Signaling Technology), phospho-mixed lineage kinase domain-like protein (37333S, P-MLKL; monoclonal (D6E3G), Cell Signaling Technology), cleaved Caspase-3 (9661S, polyclonal, Cell Signaling Technology), and beta-actin (sc-47778, monoclonal (C4), Santa Cruz Biotechnology, Santa Cruz, CA, USA).

6. L. 245. “…features similar…” is lacking in detail. Please expand on what the Authors mean.

Reply: As you advised, we expanded the sentence to clarify the meaning.

Line 245-247. A direct comparison of same-donor hWJ- and hPL-MSCs demonstrated that the features of same-donor hMSCs were similar, without significant differences between tissue sources.

7. L. 311. Provide the source and performance characteristics of the GROα ELISA, especially the lower limit of detection. See General comment.

Reply: We used a human CXCL1/GRO alpha ELISA kit (DGR00B). The sensitivity of this is 10 pg/ml and assay range is 31.3 - 1,000 pg/ml. Sample types allowed this kit are cell culture supernatants, serum, platelet-poor EDTA plasma, platelet-poor heparin plasma, and platelet-poor citrate plasma. Specificity is natural and recombinant human GRO-α. A cross-reactivity of < 0.5% was observed for the available related molecules. Cross-specific reactivity of < 50% was observed for the species tested. No significant interference was observed with the available related molecules.

8. Fig. 5.

Reply: As shown in Fig 5A, normal and lovastatin-treated C2C12 cells did not release GRO-α. This is because a human GROα ELISA kit was used to confirm GRO-α secretion. However, the hWJ-MSCs alone released a significant amount of GRO-α (Fig S2B).

6. PLOS authors have the option to publish the peer review history of their article ([what does this mean?](https://journals.plos.org/plosone/s/editorial-and-peer-review-process#loc-peer-review-history)). If published, this will include your full peer review and any attached files.


If you choose “no”, your identity will remain anonymous but your review may still be made public.


**Do you want your identity to be public for this peer review?** For information about this choice, including consent withdrawal, please see our [Privacy Policy](https://www.plos.org/privacy-policy).

Reviewer #1: **Yes:** Theodore P Ciaraldi

[NOTE: If reviewer comments were submitted as an attachment file, they will be attached to this email and accessible via the submission site. Please log into your account, locate the manuscript record, and check for the action link "View Attachments". If this link does not appear, there are no attachment files.]

While revising your submission, please upload your figure files to the Preflight Analysis and Conversion Engine (PACE) digital diagnostic tool, <https://pacev2.apexcovantage.com/>. PACE helps ensure that figures meet PLOS requirements. To use PACE, you must first register as a user. Registration is free. Then, login and navigate to the UPLOAD tab, where you will find detailed instructions on how to use the tool. If you encounter any issues or have any questions when using PACE, please email PLOS at <a href="mailto:figures@plos.org">figures@plos.org. Please note that Supporting Information files do not need this step.

*In compliance with data protection regulations, you may request that we remove your personal registration details at any time.* [*(Remove my information/details)*](https://www.editorialmanager.com/pone/login.asp?a=r)*. Please contact the publication office if you have any questions.*
